# Cellular Interactions in the Human Fatty Liver

**DOI:** 10.25122/jml-2019-1010

**Published:** 2019

**Authors:** Silvia Sovaila, Adrian Purcarea, Dan Gheonea, Sanziana Ionescu, Tudorel Ciurea

**Affiliations:** 1. Research Center of Gastroenterology and Hepatology, University of Medicine and Pharmacy, Craiova, Romania; 2. Internist.Ro Internal Medicine Clinic, Brasov, Romania; 3. First Surgical Clinic, Colentina University Hospital, Carol Davila Univeristy of Medicine and Pharmacy, Bucharest, Romania

**Keywords:** NAFLD, NASH, steatohepatitis, genetics, pathophysiology, natural history

## Abstract

Non-alcoholic steatohepatitis morbidity and mortality is on the rise due to the obesity pandemic. Its pathophysiology is not well understood and implies complex interactions between local hepatic cells populations, adipocytes, immune effectors that lead to hepatic lipid excess, lipotoxicity, cellular stress and inflammation, as well as programmed cell death. A better understanding of these pathogenic interactions would allow better identification of therapeutic targets in a disease that has no known pharmacological therapy until now.

## Introduction

### Insulin resistance, NAFLD, NASH and cirrhosis

Non-alcoholic steatohepatitis (NASH) became the leading cause of liver transplantation in a world afflicted by obesity epidemics. It is a component of the metabolic syndrome (hypertension, obesity, dyslipidemia, ectopic fat deposits) and, alongside simple steatosis, is one of the faces of non-alcoholic fatty liver disease (NAFLD).

If steatosis is the most widely met manifestation of NAFLD and represents a benign excess of fat within the hepatocyte that seldom leads to complications, NASH implies not only steatosis but also inflammation that in some patients is associated with progressive fibrosis, cirrhosis and occasionally hepatocellular carcinoma. This process seems to be driven by lipotoxicity and cellular metabolism dysfunctions caused by insulin resistance and eventually advances to chronic apoptosis, fibrosis, and cirrhosis. From hepatocytes and immune cells to adipose tissue and endothelium, multiple cellular actors are involved in NASH progression. Their action is modulated by genetic predisposition and environmental factors like diet and intestinal microbiota.

A better understanding of these pathogenic interactions would allow better identification of therapeutic targets in a disease that, until now, has no known pharmacological therapy. This review focuses on how different cells interact and mediate NASH progression.

### From fat storage to steatohepatitis

Excess fat, especially triglycerides, stored as droplets within the hepatocytes, seems to be continuously found in both steatosis and NASH. Since triglycerides are probably not hepatotoxic and even protective for the hepatocyte [1], the simple storage excess found in steatosis should not, by itself, explain the progression to NASH and fibrosis and cirrhosis [2]. For example, oleate and other monosaturated and polyunsaturated fatty acids (FAs) are associated with triglyceride-rich droplets production that corresponds to simple steatosis and are considered protective as to NASH through direct triglyceride incorporation. NASH pathogenesis is thought to be related to potentially toxic lipid moieties like saturated fatty acids (SFA), diacylglycerols [3], ceramides and sphingolipids [4-6]. Another lipid particle that might be associated with progression to NASH is cholesterol that can cause precipitation into crystals within the hepatocyte. Its exact role is still under scrutiny [6].

These hepatic fat stores originate either from dietary esterified chylomicrons or, more often, from free fatty acids (FFA). These FFAs can be either the results of a spillover mechanism where they are synthesized from lipolysis of chylomicrons by lipoprotein lipase activation or from a de novo hepatic non-lipid source production mechanisms [7,8]. They can be delivered in excess to the liver from the adipose tissue through a defective inhibition of hormone-sensitive lipase and increased adipose lipolysis promoted by insulin resistance. Within the hepatocyte, insulin resistance is directly induced by some lipid metabolites - mainly SFA and diacylglycerols - and manifests through a deficit of lipolysis suppression and an excess of de novo lipogenesis through a protein kinase Cε mediated mechanism [9]. An excess of lipids and probably lipotoxic compounds production ensues. Inversely, monosaturated and polyunsaturated FFAs do not seem to induce or aggravate insulin resistance.

At some point, the liver fatty acids excess seems to overcome the protective mitochondrial beta-oxidation and triglyceride production mechanisms from the endoplasmic reticulum and induces a lipotoxic state through metabolic stress with progressive mitochondrial dysfunction [10] and the generation of reactive oxygen species [11].

Other mitochondrial and nonmitochondrial (peroxisome, microsome) enzymatic processes, both toxic and protective, are impaired through long-chain saturated fatty acids like palmitate and stearate excess and seem to participate in this cellular stress state [12-14].

Reactive oxygen species and excess lipotoxins within the hepatocyte lead to the saturation of the endoplasmic reticulum, which responds through a stress state of unfolded protein response (UPR) [15]. This state is characterized by decreased protein and lipid secretion, lipolysis, and initially increased autophagy, which, in the short term, is meant to protect the hepatocyte [16]. In the long term, oxidative stress, NF-kB-mediated inflammatory response [17], insulin resistance [18] and programmed cell death [17,19] are progressively upregulated by the stressed endoplasmic reticulum and perpetual UPR state and become deleterious [20]. This positive loop process leads to hepatic inflammation, autophagy, and repeated programmed cell death and represents the main driver for the passage from simple fatty liver to NASH and, ultimately, cirrhosis.

Autophagy is upregulated in NASH by an excess of unfolded proteins and excess lipid droplets through the mTOR and PI3K pathways, but clearance of the substrate is insufficient. Defective autophagy could explain the persistence of lipotoxic moieties and inflammation within the hepatocyte, which finally leads to programmed cell death and increased hepatic insulin resistance [21]. Insulin resistance independently increases apoptosis through the PI3K, ERK, and MAPK pathways [22] but also through reactive oxygen species generation and mitochondrial permeabilization [23].

Pyroptosis, another type of programmed cell death tied to NF-kB activation, is excessive in individuals with NASH via an excess of gasdermin D signaling that correlates linearly with NAFLD activity and induces a pro-inflammatory environment within the liver [24]. These lipotoxic pathways of inflammation and programmed cell death for the hepatocyte are similar to those that induce activation and fibroblast transformation of hepatic stellate cells [25].

Since lipid storage alone does not always lead to steatohepatitis, factors like genetics [24], microbiota – host interactions, and impaired immune response probably contribute to NASH progression. Specific mitochondrial protein-coding genes [26] but also genes involved in collagen and cholesterol synthesis (Patatin-like phospholipase domain-containing protein 3 - (PNPLA3) [27], insulin signaling (Insulin Receptor Substrate 1 - IRS1), and innate immunity activation (MERTK) inconstantly and heterogeneously associate with NASH. Other external factors like age and nutrient intake (fat or sugars) are important modifiers of the steatosis phenotype; it is accepted that we need to account for extensive interactions with the genotype if we are to model the disease progression [28].

Another target that could explain phenotypic differences in the fatty liver is the host-microbiota interaction. Disturbance of the gut–liver axis by inflammation or bile acid signaling could initiate or aggravate NASH, maybe through excess bacterial lipopolysaccharides within the portal vein. Lipopolysaccharides exposition induces inflammation and autophagy within the healthy liver, but the causal association with NASH is still to be determined.

### Inflammation, hepatocyte death and stellate cell activation and differentiation

Until this point, steatohepatitis is mainly confined to the hepatocyte cytoplasm. Once the endoplasmic reticulum is overwhelmed by lipotoxic by-products and UPR is initiated, and mitochondrial dysfunction with reactive oxygen species formation alongside insulin resistance activates inflammation and programmed death, the process spreads to the nucleus and outside the hepatocyte. Inflammation is initiated by Fas, NF-Kb and TNF receptors, as well as TNF-related apoptosis-inducing ligand receptor [23]. Hepatocyte destruction releases damage-associated molecular patterns (DAMPs) and induces reactive oxygen species that further increase activation of hepatic stellate cells [29] and perpetuates the inflammatory milieu. Through this signaling cascade, macrophages and Kupfer cells residing the liver are recruited and initiate the innate immune response [30,31]. They keep the principal functions, such as phagocytosis, danger signal recognition, cytokine release, antigen processing, and the ability to modulate immune responses [32] but exposure to endotoxins, pathogen-associated molecular pattern (PAMPs), lipopolysaccharides and specific FFAs and cholesterol metabolites limit macrophage differentiation towards fibrinolytic phenotypes [33]. Alongside Kupfer cells and macrophages, activated resident natural killer (NK) cells and natural killer T (NKT) cells produce cytokines that also mediate lymphocyte recruitment [34] and DAMP and PAMP signaling that amplifies apoptosis. In a cascade, pyroptosis continues and cytokines (TNF, Il2, IL6, IL 17) are released, and more effector immune cells are recruited [35]. The profibrogenic effect of innate immunity activation is secondary to TGF-beta secretion by most residing cells (hepatocytes, immune cells, macrophages), which stimulates the transformation of stellate cells into myofibroblasts [36]. Activated endothelial cells mediate this process through the expression of adhesion molecules that facilitate neutrophil and lymphocyte recruitment [37]. Their secretion of IL33 and IL1 superfamily cytokines stimulates the activation of perisinusoidal stellate cells and explains perisinusoidal fibrosis [38]. High titers of antibodies against lipid peroxidation-derived antigens [39] and activated lymphocytes [40] parallel with parenchymal infiltration by lymphocytes suggest the role of adaptative immunity in the perpetuation of NASH and progression to cirrhosis. Th1/Th2 CD4 + T helper lymphocytes population ratio immunomodulates fibrosis evolution via cytokine synthesis [41]. B and T cell infiltrates correlate with more severe lobular inflammation [42].

This inflammatory process leads to the activation of the main effector of fibrosis progression, the hepatic stellate cells. They represent less than 5% of the healthy liver population that are generally found in the space of Disse and have a role in the storage of lipids and fat-soluble vitamins [43]. Their activation and proliferation modulated by local inflammatory cytokines [31,44, 45,46], lipoprotein lipase pathway [47], circulating noncoding RNA (microRNA and long non-coding RNA) [48,49] and growth factors lead to fibrosis and cirrhosis. When activated, hepatic stellate cells express alpha-smooth muscle actin and transform into myofibroblast. Upon activation, they display a necro-inflammatory activity [50], immunomodulatory, and metalloproteases (TMP1) inhibitory role. They promote fibrosis via extracellular type I and III collagen synthesis [51] and are stimulated by locally produced factors comprising transforming growth factor-beta (TGF-β) and platelet-derived growth factor (PDGF). These myofibroblasts are currently considered to have a central role in liver fibrosis progression [52]. In a minority of patients suffering from NASH, this process will perpetuate, and severe fibrosis and cirrhosis will ensue.

## Conclusion

Steatohepatitis is a complex and heterogeneous syndrome in which insulin resistance and impaired lipid metabolism seem to induce a constant state of hepatic inflammation that leads to hepatocyte programmed death and activation of tissue repair mechanisms that will eventually conduct to fibrosis and cirrhosis.

## Conflict of Interest

The authors confirm that there are no conflicts of interest.
